# Tracking Self-Control – Task Performance and Pupil Size in a Go/No-Go Inhibition Task

**DOI:** 10.3389/fpsyg.2022.915016

**Published:** 2022-06-07

**Authors:** Sinika Timme, Wanja Wolff, Chris Englert, Ralf Brand

**Affiliations:** ^1^Sport and Exercise Psychology, University of Potsdam, Potsdam, Germany; ^2^Department of Sport Science, Sport Psychology, University of Konstanz, Konstanz, Germany; ^3^Institute of Educational Science, University of Bern, Bern, Switzerland; ^4^Institute for Sports Sciences, Goethe University Frankfurt, Frankfurt, Germany

**Keywords:** self-control, response inhibition, psychophysiological, behavioral and self-report measures, pupil diameter

## Abstract

There is an ongoing debate about how to test and operationalize self-control. This limited understanding is in large part due to a variety of different tests and measures used to assess self-control, as well as the lack of empirical studies examining the temporal dynamics during the exertion of self-control. In order to track changes that occur over the course of exposure to a self-control task, we investigate and compare behavioral, subjective, and physiological indicators during the exertion of self-control. Participants completed both a task requiring inhibitory control (Go/No-Go task) and a control task (two-choice task). Behavioral performance and pupil size were measured during the tasks. Subjective vitality was measured before and after the tasks. While pupil size and subjective vitality showed similar trajectories in the two tasks, behavioral performance decreased in the inhibitory control-demanding task, but not in the control task. However, behavioral, subjective, and physiological measures were not significantly correlated. These results suggest that there is a disconnect between different measures of self-control with high intra- and interindividual variability. Theoretical and methodological implications for self-control theory and future empirical work are discussed.

## Introduction

Self-control refers to “the set of mechanisms required to pursue a goal, especially when distraction and/or strong (e.g., habitual) competing responses must be overcome” (Shenhav et al., 2013, p. 217). For example, self-control needs to be applied if one wants to inhibit an impulse or resist a temptation that would be at odds with one’s ongoing goal pursuit (e.g., Fujita, 2011). In light of the importance of self-control in reaching valued goals, a variety of theories and models have been proposed to explain why self-control can sometimes fail (e.g., Kurzban et al., 2013; Shenhav et al., 2013; Wolff and Martarelli, 2020). There are more than a few controversial issues to be resolved, however: One is that only little is known about the psychological and physiological processes involved during the implementation of self-control. Therefore, the aim of this study is to investigate the temporal dynamics and potential interactions of presumably relevant subjective, behavioral, and physiological markers that have been associated with self-control exertion in prior research.

In the last two decades, research on self-control has been shaped by hypotheses derived from the highly popular strength model of self-control (Baumeister et al., 1998). Although a large body of research initially supported the strength model (Hagger et al., 2010), its validity has been questioned in recent years on empirical as well as theoretical grounds (e.g., Beedie and Lane, 2012; Lee et al., 2016). While some criticism refers to the mechanistic underpinning (Beedie and Lane, 2012), specific criticism has been directed toward the experimental setups that have traditionally been used in self-control research, in particular what is known as the sequential two-task paradigm (e.g., Lee et al., 2016). In this paradigm, participants are requested to first work on a task that is thought to require high levels of self-control (i.e., experimental condition) or a similar task that presumably requires close to no self-control (i.e., control condition). Then a second task follows, in which each participant has to invest self-control in order to succeed. Participants whose self-control strength has been experimentally reduced with the first task (“depletion task”) are expected to perform worse on the second task. While this paradigm can detect carry-over effects, the true cause of the observed performance impairment still remains unclear. Early meta-analytic evidence has suggested that the sequential two-task design can reliably induce results that are consistent with the strength model’s predictions (i.e., impaired performance on a secondary task if preceded by a primary self-control-demanding task) (Hagger et al., 2010). However, evidence for publication bias and large-scale pre-registered replication failures of the sequential two-task paradigm have accumulated in recent years (e.g., Hagger et al., 2016; Wolff et al., 2018).

Against this background, Arber et al. (2017) examined the previously untested assumption that performance declines over time on tasks that are assumed to “deplete” self-regulatory resources. The authors (Study 4) showed as hypothesized that the number of errors participants made while completing a letter-crossing task (Baumeister et al., 1998; Hagger et al., 2010) increased the longer participants worked on it.

Although participants report feeling less vital and energized after self-control demanding tasks, such as in measures of subjective vitality (Forestier et al., 2018; Bertrams et al., 2020), this may not translate directly into impaired performance (Wolff et al., 2019). Inconsistent findings such as these limit our understanding of the psychological concept of self-control. Accordingly, researchers have called for using more fine-grained experimental approaches in order to investigate the temporal dynamics of a more comprehensive set of self-control measures and markers (Friese et al., 2019; Wolff and Martarelli, 2020). Analyzing the association of different measures and their temporal dynamics may enhance insights about underlying mechanisms of self-control application and thus contribute to the current debate regarding the conceptualizing of self-control.

Due to its association with processes involved in self-control and its relative direct response compared to other autonomic measures (reacts in response to stimuli in 0.2–0.3 s and peaks in about 0.5–1.0 s), previous research has suggested pupil dilation as a possible biometric marker to track changes in self-control (e.g., Friese et al., 2019). However, since there has been no research on this topic in the field of self-control research yet, findings from relevant related areas are presented below and working hypotheses are formulated, which can, therefore only be of explorative nature.

It is known that the application of self-control is effortful and aversive (Kool and Botvinick, 2013). The exertion of the effort required to perform an act of self-control can lead to sensations of fatigue (Wolff et al., 2019). These processes have been shown to be related to changes in pupil diameter. There is ample evidence of stimulus-evoked increases in pupil diameter during effortful cognitive control, such as when executive functions like inhibition, updating, and shifting are tested (van der Wel and van Steenbergen, 2018). On the other hand, it has been long known that pupil diameter decreases with fatigue and reaches a minimal diameter before falling asleep (and increases in states of high alertness) (Lowenstein and Loewenfeld, 1964). Additionally, a series of recent experiments, Hopstaken et al. (2015, 2016) demonstrated that baseline pupil diameter decreased during a fatiguing experimental task. Interestingly, it was restored when participants were offered the prospect of a motivating reward. These findings have their basis in neurological mechanisms, as changes in pupil diameter have been shown to be a sensitive psychophysiological measure of activation in the autonomic nervous system (ANS). Whereas an activation of the sympathetic system (e.g., during mental effort) induces widening of the pupil (dilation), neurons of the parasympathetic pathway induce pupil constriction (Andreassi, 2000). Hopstaken et al. (2015) have also looked at the relationship between subjective (fatigue), behavioral (performance) and psychophysiological measures (P3 and pupil dilation) during a mentally fatiguing task. Their findings support the relationship between these measures. It must be noted, though, that the findings do not yet paint a consistent picture (van der Wel and van Steenbergen, 2018). Therefore, the aim of our study is to model the changes in pupil diameter over time in an exploratory manner and examine its association with a subjective and a behavioral measure of self-control.

To address this issue, this study aimed to continuously track the temporal progression of a behavioral, subjective, and psychophysiological measure in two tasks with different self-control demands (inhibition task: high vs. control task: low). Additionally, the association of these measures was examined. First, we expected to track a hypothesized decline in performance that occurs during an inhibition task requiring self-control on the behavioral level. Second, the performance measure was compared with participants’ reported feeling of vitality before and after the task. We expected subjective vitality to decrease from before to after the tasks and to be associated with impaired performance. Third, we monitored changes in pupil diameter during the tasks, expecting that those changes would be associated with the performance measure. Fourth, we wanted to explore the possible correlation between changes in pupil diameter and self-reported subjective vitality. To account for inter- and intraindividual variability, mixed modeling approaches with fixed and random factors were used to analyze the data.

## Materials and Methods

### Experimental Design and Participants

We used a within-subject experimental design, with each participant undergoing both experimental and control conditions. Subjects with neurological or psychiatric conditions, severe limitations in sight, varifocal glasses, sunglasses, or polarized glasses (simple glasses and contact lenses were accepted) were not eligible to participate. Due to the lack of previous research and theory to accurately specify all components of the mixed models, we refer to simulation studies that suggest that at least 50 level-2 units (i.e., participants) are necessary to accurately estimate standard errors in mixed effects modeling (Maas and Hox, 2005).

We recruited a group of 65 university students (*M*_*age*_ = 24.2, SD_*age*_ = 3.25, 65.6% female). Behavioral data from 5 participants had to be excluded (*n* = 3 in the experimental condition, and *n* = 3 in the control condition because they had more than 75% errors, indicating non-compliance with the instructions). This resulted in a sample of 62 study participants for the behavioral data analysis in the experimental condition (*M*_*age*_ = 24.14, SD_*age*_ = 3.24, 66.1% female) and 62 in the control condition (*M*_*age*_ = 24.23, SD_*age*_ = 3.32, 66.1% female). Additionally, eye-tracking data from 7 participants (*n* = 4 in the experimental condition, *n* = 3 in the control condition) had to be excluded due to recording malfunction (*n* = 4) or to behaviors reported by the participant that may have had a severe impact on the ANS prior to the experiment (*n* = 3, e.g., intense physical activity immediately before the study). This resulted in a sample of 58 study participants for the pupil data analysis in the experimental condition (*M*_*age*_ = 23.94, SD_*age*_ = 3.10, 65.5% female) and 59 in the control condition (*M*_*age*_ = 24.16, SD_*age*_ = 3.37, 67.8% female). All participants gave their full informed consent prior to the study and formally accepted the data sharing regulations and privacy policies. Data were stored anonymously according to the EU General Data Protection Regulation (GDPR). All procedures were conducted in compliance with the Declaration of Helsinki and ethical guidelines of the American Psychological Association (APA).

### Variables and Equipment

Test presentation and performance data acquisition was performed using the Inquisit (2016 edition) software. Pupil data acquisition and analysis were performed in the iMotion™ 8.1 platform for biometric research.

#### Experimental Condition (Inhibition Task)

A Go/No-Go task presented on a computer screen was used to measure inhibitory control. Earlier studies used similar tasks to investigate self-control (e.g., Lange et al., 2014). Here, participants were instructed to respond to the letter “X” as quickly as possible by pressing the space bar on the computer keyboard, and to withhold this response when the letter “Y” appeared. Each stimulus was presented for 500 ms, followed by a 500-ms interstimulus interval (blank screen). Response accuracy (correct vs. incorrect) was recorded for keystrokes on all trials. The omission of a response after Go-trials was recorded as an “incorrect” response. If the response could be withheld after inhibition trials (*y*-trials; No-Go trials), “correct” was recorded, but if the participant showed a response, “incorrect” and the response time were registered. Response time was not considered in the analysis, as “correct” inhibition responses required no reaction and thus produced equal response times (i.e., the 500-ms stimulus presentation time). Participants did not receive feedback on response accuracy. The task consisted of 600 trials, and the ratio between inhibition trials (*y*) and Go-trials (*x*) was 1:4 in randomized order. This was done to establish pressing the space bar as the dominant (i.e., normally required) response to stimulus presentations and thus make the required inhibition of a response to *y*-trials more difficult (see Baumeister and Vohs, 2016, for the underlying rationale). There is evidence that rare No-Go trials evoke prepotent motor activity requiring inhibitory control (Wessel, 2018).

The task was displayed on the screen with a minimalist layout (i.e., white screen with uppercase black letters in Arial font size, with 10% letter height relative to screen height). Participants were instructed to respond to trials “as quickly as possible.” Total test duration was about 10 min.

#### Control Condition (Two-Choice Reaction Time Task)

For the control condition, the Go/No-Go task described above was modified into a Go/Go task, that is, a two-choice reaction time task (Gomez et al., 2007). Here, the same stimuli (“X” and “Y”) were used, but both required a keypress (“E” or “I” on the keyboard). Response buttons were counterbalanced between participants (“E” for an *x*-stimulus and “I” for a *y*-stimulus, and vice versa). Both response time and response accuracy were recorded. The rest of the procedure imitated that of the inhibition task. In the control task, neither response (ratio 1:1) is more difficult than the other. The two-choice task was chosen because it does not promote the development of a dominant prepotent motor response (Wessel, 2018). Thus, the control task differed from the inhibition task in that commission errors as indicative of inhibitory control failure would not be possible.

#### Subjective Vitality

Participants were asked to respond to the two items “I feel alive and full of vitality” and “I feel I have a lot of energy” from the Subjective Vitality Scale (Ryan and Frederick, 1997). The answer scale ranged from 1 (“completely disagree”) to 7 (“completely agree”). This scale has been used to measure subjective state self-control in previous studies (e.g., Forestier et al., 2018) and has shown strong correlations with self-report and performance measures of self-control (Martela et al., 2016; Bertrams et al., 2020).

#### Pupil Diameter

Pupil size in both eyes was continuously measured at a sampling rate of 64 Hz with the Gazepoint™ GP3 infrared eye-tracking system. Lighting conditions and the stimuli luminance were held constant during all measurements. The eye-tracker was placed 65 cm in front of the participants, beneath the computer screen (Benq Senseye FP222Wa, 22”) where the tasks and questions were presented.

### Procedure

Subjects were tested individually. After participants had read the general information about study participation and consented, they sat down in a single-seat cabin with LED light strips attached to the walls for constant illumination, and a computer monitor in front of them. Participants were randomly assigned to complete either the inhibition task or the control task during their first lab session. The experimenter began the study, and the participant followed the instructions on the screen. After successful calibration of the eye tracker, the participants responded first to the subjective vitality measure. Afterward, they performed either the inhibition task or the control task, and were then asked to answer the questions on subjective vitality again. Finally, the participants were debriefed and thanked for their participation in the study. At the end of this first session, participants were given an appointment for their second lab visit for the following week. The procedure for the second lab visit was identical to the first one, except that the other task (the control task instead of the inhibition task, or vice versa) was completed.

### Statistical Analysis

Mixed-effects models were used for statistical analysis with the *lme4* package (Bates et al., 2015) in the R statistical computing environment (R Core Team, 2019). The *lmerTest* package (Kuznetsova et al., 2017) was used to calculate Satterthwaite’s approximation for degrees of freedom.

#### Task Performance (Error Analysis)

Error rate was used as the *performance* measure in the inhibition task and the control task. In both tasks, there were two *trial types* (*x*- and *y* trials) for which error rates were calculated. Errors in withholding the response to *y*-trials in the inhibition task (i.e., in inhibition trials: inhibitory control failure) were referred to as *commission errors*. Omitted responses to *x*-trials were *omission errors*. Pressing the wrong key or missing the response to the stimulus in the control task (“E” instead of “I” or “I” instead of “E”) was either an *x-error* or a *y-error* depending on the displayed stimulus. For the statistical analysis, the 600 trials were segmented into *blocks* 1–6. For each block, the number of errors was divided by the number of trials, resulting in the proportion of errors per block (error rate). A Poisson generalized linear mixed model for *performance* with the within-predictors *task* (inhibition vs. control), *trial type*, and *block* (change over time) was calculated to analyze the data. *Post hoc* contrasts were applied in order to compare performance between blocks.

#### Subjective Vitality

Change in subjective vitality from before to after the task was analyzed using a linear mixed model with the within-predictors *task* and *time* (before vs. after completion of the task).

#### Pupil Diameter

Pupil diameter was calculated by averaging the diameters of the right and left pupils. Similar to the error analysis, the 600 trials in both tasks were segmented into six blocks. The average pupil size for each block was calculated. Artifacts and blinks were detected by the eye tracker and removed from the analysis. Pupil diameter before participants began working on the task was included in the model to account for possible differences in baseline pupil diameter (pre-start). Baseline pupil diameter was recorded while participants viewed a blank screen without stimulus presentation. A linear mixed model for pupil diameter with the within-predictors *block* and *task* was calculated for the analysis. *Post hoc* contrasts were applied in order to compare pupil size between blocks.

## Results

### Performance

The mixed model for *performance* and the three predictors *trial type*, *block*, and *task* revealed a significant three-way interaction (*b*_block*trial*task_ = 0.034, *p* < 0.001). This means that the performance changed from block to block, but this change was different in the two tasks and for the different types of errors. To disentangle the three-way interaction, *performance* was regressed on *trial type* and *block* for both tasks (inhibition and control) separately in the next step. Full model parameters and the corresponding Incidence Ratio Rates can be found in Table 1.

**TABLE 1:** Parameter estimates of the performance (error rates) in separate models for the inhibition and the control task.

	Inhibition task			Control task		
	**IRR**	**95% CI**	***p***	**IRR**	**95% CI**	***p***
	
**Fixed effects**						
Intercept	9.44	8.22–10.84	< 0.001	23.86	21.29–26.73	< 0.001
Block	1.03	0.99–1.06	0.11	1.00	0.98–1.02	0.79
Trial	2.43	2.09–2.82	< 0.001	1.01	0.98–1.04	0.59
Block * Trial	1.06	1.02–1.09	< 0.01	0.99	0.98–1.00	0.02

**Random effects**						
Residual		0.07			0.04	
Intercept		0.29			0.20	
Block		0.01			0.00	
Trial		0.35			0.01	
Block * Trial		0.01			0.00	

Conditional *R*^2^		0.96			0.85	

*CI, Confidence Interval; IRR, Incidence Rate Ratios, which are calculated as the exponent of the regression estimates.*

#### Inhibition Task

**FIGURE 1:**
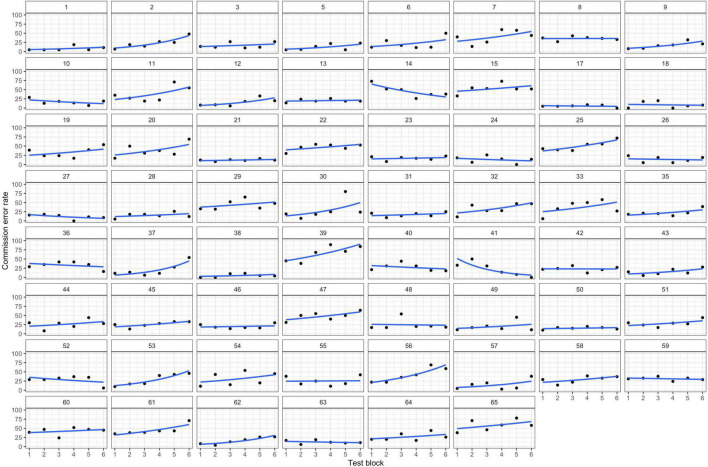
Individual Trajectories of Inhibitory Control Performance. The black dots are the individuals’ commission error rates (number of errors divided by number of trials) in each block of the inhibition task. The blue lines display the individual linear slopes of inhibitory performance across blocks. Increasing slope signals decreasing inhibitory control.

First, we regressed *performance* on *block* and *trial type* with random intercepts for participants. Introducing random slopes for *block*, *trial type*, and their interaction significantly improved the model fit, χ^2^ (9) = 2423.8, *p* < 0.001, indicating that there was significant interindividual variability in participants’ performance across *blocks* and *trial type*. Figure 1 displays the observed data with predicted individual trajectories.

There were significant effects for *trial type* (*b*_*trial*_ = 0.887, *p* < 0.001) and the interaction of *block* with *trial type* (*b*_block*trial_ = 0.054, *p* < 0.001). This indicates that the change in performance was different for commission and omission errors. Specifically, the regression parameters and the resulting Incidence Rate Ratios (see Table 1) revealed that commission errors increased by 8.4% with each block, while omission errors decreased just marginally by 2.7%. Block-wise comparisons revealed significant increases in commission errors from blocks 1 to 6 (*b*_1–6_ = 0.407, *p* < 0.001), with the largest increase from blocks 2 to 3 (*b*_2–3_ = 0.16, *p* = 0.07). All other subsequent block contrasts showed a small, but non-significant increase in commission errors. There was no significant increase in omission errors from blocks 1 to 6 (*b*_1–6_ = 0.028, *p* = 0.99).

#### Control Task

There was no significant effect for *block* (*b*_*block*_ = −0.002, *p* = 0.79) or *trial type* (*b*_*trial*_ = 0.009, *p* = 0.59), but a significant albeit small interaction effect (*b*_block*trial_ = −0.014, *p* = 0.02). Quantifying the interaction effect for *x*- and *y*-error slopes revealed a small predicted linear increase of 1.1% for *x*-errors and a slightly different decrease for *y*- errors of 1.6%. Block-wise comparisons revealed that the only significant change in performance was a small increase from blocks 3 to 4 for *x*-errors (*b_3–4_* = 0.155, *p* = 0.05). No other differences between blocks (*b* = −0.157–0.140, *p* = 0.25–0.99) or trial types (*b* = −0.121–0.009, *p* = 0.06–0.85) were significant.

**FIGURE 2:**
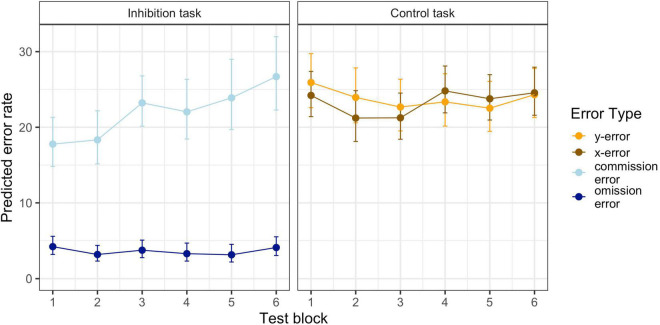
Predicted Mean Performance on the Inhibition and the Control Tasks in the Six Test Blocks. Error bars represent standard errors.

Summarizing the results from the inhibition and control task indicates a decrease in inhibition performance (successively more commission errors), whereas the number of any other error remains constant. Figure 2 displays the predicted model-based cumulative effects for all error types across blocks. Full model parameters can be found in Table 1.

### Subjective Vitality

**FIGURE 3:**
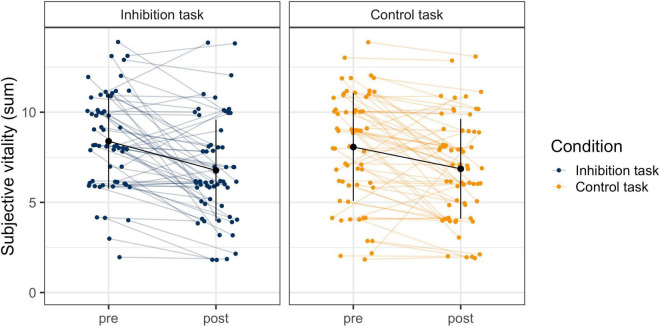
Change in Subjective Vitality Before and After the Inhibition and the Control Task. The blue and orange dots are the individuals’ raw data from before and after the tasks, which are connected with the colored horizontal lines. The black dots and vertical lines display the mean and standard deviation at each timepoint in each task.

The model for subjective vitality with the two predictors *time* and *task* and random slopes provided a significantly better model fit than the model with random intercepts only, χ^2^ (5) = 18.59, *p* < 0.01. The model with random slopes was therefore used for the final statistical analysis. There was a significant effect for *time* (*b*_*pre–post*_ = −1.401, *p* < 0.001), but not for *task* (*b*_*task*_ = −0.159, *p* = 0.43) or the interaction (*b*_post*task_ = 0.191, *p* = 0.36). This means that participants reported a significant and comparable decline in subjective vitality in both tasks, but with significant individual variability in their slopes (see Figure 3).

#### Subjective Vitality and Performance

To examine the relationship between performance and subjective vitality, change in performance (predicted slope of error rate) was correlated with change in subjective vitality (pre- to post-task). Additionally, performance at the end of the task (number of errors in block 6) was correlated with subjective vitality after the task. There was no significant correlation of either inhibitory performance or any other performance measure (omission errors, *x*- and *y*-errors on the control task) with subjective vitality in their rate of change (inhibitory task: *r*_*y*_ = −0.12, *p* = 0.35; *r*_*x*_ = 0.08, *p* = 0.56; control task: *r*_*y*_ = −0.12, *p* = 0.35; *r*_*x*_ = −0.01, *p* = 0.92). Similarly, performance at the end of the task was not related with vitality after the task (inhibitory task: *r*_*y*_ = −0.22, *p* = 0.10; *r*_*x*_ = −0.13, *p* = 0.33; control task: *r*_*y*_ = −0.02, *p* = 0.88; *r*_*x*_ = −0.10, *p* = 0.44). This means that the behavioral measures for self-control varied independently of the subjective measure.

### Pupil Size

**FIGURE 4:**
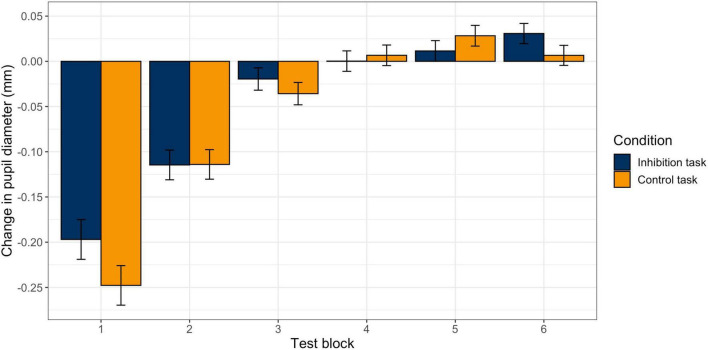
Changes in Pupil Diameter between Blocks in the Inhibition and the Control Task. Estimated change in mean pupil diameter from block to block in the inhibition (blue) and control (orange) tasks. Error bars represent standard errors.

The model for pupil size with the predictors *block* (change over time) and *task* (inhibition vs. control) with random slopes for *block* and *task* provided a significantly better model fit than the model with random intercepts only, χ^2^ (5) = 152.13, *p* < 0.001. Therefore, the model with random slopes was used for the final statistical analysis. There was a significant effect for *block* (*b* = −0.04, *p* < 0.001), but not for *task* (*b* = −0.003, *p* = *0.79*), and no significant interaction effect (*b* = 0.004, *p* = *0.09*). This indicates that there was a significant and similar decrease in pupil diameter during the two tasks. Figure 4 visualizes the change in pupil size across blocks in the two tasks. The exact values can be found in Table 2. First, pupil diameter decreases continuously until block 3. Block-wise comparisons revealed that there was a significant decrease from pre-start to block 1 (inhibition task: *b*_0–1_ = −0.20, *p* < 0.001; control task: *b*_0–1_ = −0.25, *p* < 0.001) and from block 1 to block 2 (inhibition task: *b*_1–2_ = −0.11, *p* < 0.001; control task: *b*_1–2_ = −0.11, *p* < 0.001) in both tasks. In blocks 3 and 4, the pupil diameter remains reduced. Then, in block 5, there are signs of an increase in pupil size (with the pupil still small), that further have developed in block 6, especially in the inhibition task (*b*_5–6_ = 0.03, *p* = 0.02; control task: *b*_4–5_ = 0.03, *p* = 0.04). There were no significant differences in pupil size between the two tasks in any of the blocks.

**TABLE 2:** Observed means for pupil diameter across blocks for the two tasks separately.

Block	Inhibition task		Control task	
	
	*M*	SD	*M*	SD
Start/pre-task	3.66	0.47	3.74	0.51
Block 1	3.45	0.39	3.49	0.47
Block 2	3.34	0.33	3.36	0.37
Block 3	3.32	0.32	3.33	0.35
Block 4	3.31	0.32	3.34	0.34
Block 5	3.32	0.31	3.36	0.35
Block 6	3.35	0.34	3.36	0.36

#### Pupil Size and Performance

To examine the relationship between performance and pupil size, change in performance (predicted slope of error rate) was correlated with change in pupil size (predicted slope of pupil size). There was no significant correlation of either inhibitory performance or any other performance measure (omission errors, *x*- and *y*-errors in the control task) with pupil size in their rate of change (inhibition task: *r*_*y*_ = 0.05, *p* = 0.71; *r*_*x*_ = 0.07, *p* = 0.62; control task: *r*_*y*_ = −0.05, *p* = 0.73; *r*_*x*_ = 0.12, *p* = 0.41). This means that the behavioral measures for self-control varied independently of the physiological measure.

#### Pupil Size and Vitality

To examine the relationship between subjective vitality and pupil size, change in subjective vitality (pre- to post-task) was correlated with change in pupil size (predicted slope of pupil size). There was no significant correlation of change in subjective vitality with pupil size in any task (inhibition task: *r* = 0.13, *p* = 0.34; control task: *r* = −0.11, *p* = 0.45). This means that the subjective measures for self-control varied independently of the physiological measure.

## Discussion

This study tracked subjective, behavioral, and physiological indicators of self-control exertion during tasks that were designed to pose different self-control demands. Over the course of the self-control-demanding task (Go/No-Go task), we observed a decrease in inhibition performance, which was tracked as a behavioral marker of self-control performance. In the control task, where no habitual response was developed and therefore posed lower self-control demands, no change in performance was observed. This indicates that the demands to apply inhibitory control increased more and more in the Go/No-Go task while they remained rather constant in the control task.

In contrast, subjective vitality decreased in both tasks and this decrease did not differ between tasks. Focusing solely on the self-report measure could have led to the conclusion that both tasks reduced a self-control resource. Likewise, trajectories of change in pupil diameter were similar in both tasks. The pupil decreased during the first two blocks, then remained constant until about block 5 and increased slightly toward the end. Based on the similar trajectories, it can be assumed that similar processes underlying pupil dilation (e.g., effort, fatigue, learning) occurred in the inhibition and the control task.

Taken together, tasks that appear to affect psychological (i.e., subjective vitality) and physiological indicators (i.e., pupil dilation) of self-control exertion in a similar fashion differ with respect to their level of task-imposed self-control demands. Thus, our results point toward a disconnection between structural, task-imposed self-control demands (i.e., high vs. low inhibitory control) on the one hand and subjective self-control costs (as operationalized by experienced subjective vitality) on the other hand, as well as the temporal dynamics of pupil dilation during task completion (psychophysiological level). This disconnect is hard to reconcile with the notion of a singular, limited self-control resource (Baumeister et al., 1998). Instead, our findings appear to be more in line with recent theories that avoid the notion of a limited self-control resource and instead conceptualize self-control application as the result of a reward-based choice (e.g., Kurzban et al., 2013; Shenhav et al., 2013, 2017).

In light of mounting criticism regarding the strength model’s mechanistic underpinnings (e.g., Beedie and Lane, 2012; Lee et al., 2016) and repeated, large-scale failures to replicate its main premises (e.g., Hagger et al., 2016; Vohs et al., 2021), more recent theorizing has shifted from resource-based accounts of self-control (Baumeister et al., 1998) to understanding self-control as a reward-based choice (Shenhav et al., 2013). This implies that performance on a self-control-demanding task is impaired because a person is *unwilling* rather than *unable* to apply the required control (Englert et al., 2020). This unwillingness is thought to be driven by the anticipated costs that accompany prolonged application of self-control, which biases the cost-benefit analysis away from exerting further self-control (Shenhav et al., 2017; Wolff and Martarelli, 2020). While the exact nature of these costs is still up for debate – whether they are intrinsic to self-control (Kool and Botvinick, 2013) and/or reflect opportunity costs (Kurzban et al., 2013) – empirical and theoretical work converges in that application of self-control application is aversive, and humans (and non-human animals) prefer to avoid incurring these costs (Gieseler et al., 2020). In turn, self-control is only applied if the expected value of the action (e.g., getting course credit for participating in a psychology experiment) outweighs the costs it incurs (e.g., the effort to perform the experimental task correctly) (Shenhav et al., 2017).

Referring to the present study, why did we observe an increase in participants’ error rate in the self-control-demanding Go/No-Go task but no task-dependent differences in subjective vitality after either task was completed? One tentative explanation is that both tasks demand self-control (e.g., in the form of opportunity costs), but the same amount of required effort is not sufficient to perform equally well. According to the Expected Value of Control (EVC) theory (Shenhav et al., 2013), individuals adjust the amount of self-control they allocate in any given situation in a way that maximizes the EVC. As the rewards for performing well on the task were not contingent on task performance (i.e., participants were not incentivized for good performance), the amount of effort participants could justify to deploy toward either task was likely to be the same for both tasks (and did not develop divergently across blocks as a function of task type). Indeed, subjective vitality ratings, as a proxy for the task-incurred experiential costs, and the trajectories of pupil diameter were similar for both tasks. Given no added incentive to perform well on either task, one would expect error rates to increase when the justified effort, as specified by the EVC, does not suffice to perform well. Indeed, only in the Go/No-Go task did we observe an increase in inhibitory control error rate as a function of task duration. However, this still begs the question as to why the task-imposed self-control demands changed: One potential explanation would be that a stronger prepotent motor response toward the default response (i.e., the one that was required more frequently) evolved as a function of task duration.

In addition, it has been proposed that boredom might systematically affect results in self-control research as an uncontrolled confound (Wolff and Martarelli, 2020) and research on self-control has started to emphasize the potential relevance of boredom in this context (e.g., Kurzban et al., 2013: Milyavskaya et al., 2019). But *how* is boredom – a sensation whose functional relevance for orienting behavior has only recently started to garner attention – thought to affect performance in experimental self-control research? In a nutshell, current theorizing on boredom suggests that boredom acts as a strong motivator for exploration, leading to an urge to stop what one is currently doing and to engage in something else (Danckert, 2019). During a task that requires continued responding (e.g., during a Go or a Go/no Go task), this urge must be controlled, and boredom therefore acts as an additional (and un-accounted for) self-control demand that is placed on a participant (Wolff and Martarelli, 2020).

Importantly, the experimental and control tasks that are typically used in research that is informed by the strength model framework might differ systematically with respect to how much boredom they induce, and in turn the degree to which this poses additional self-control demands (for an in depth discussion of this argument, please see Wolff and Martarelli, 2020). One reason for such differences in task-induced boredom can be found in how tasks are often designed in self-control research: To minimize the self-control demands in the control condition, this task is typically very easy and for example merely requires categorizing stimuli without the need to control an impulse and override a default response. However, these properties render such tasks almost ideally suited to induce boredom, and tasks that are used as control conditions have even been used as boredom inductions in boredom research^1^ (Wolff and Martarelli, 2020). This implies that tasks that are typically used as control tasks can also be self-control-demanding and even lead to perceptions of resource depletion and fatigue (Westgate and Wilson, 2018). Indeed, recent empirical work is consistent with the proposition that control tasks in ego depletion research can be more boring than the respective self-control demanding task (e.g., a congruent Stroop task compared to an incongruent Stroop task), and that this affects downstream performance (Bieleke et al., 2021).

Unfortunately, boredom was not measured in the present study. However, based on theoretical propositions about how boredom occurs (Westgate and Wilson, 2018), the type of tasks that are used (Markey et al., 2014), and empirical evidence on differences in boringness between control and experimental conditions in self-control research (Bieleke et al., 2021), it is plausible to assume our control task – albeit being less intrinsically demanding - was experienced as more boring. This reasoning is consistent with the drop in subjective vitality we observed in both tasks. However, this interpretation is speculative at this point and in future self-control studies, it is paramount to control for boredom to rule out this potential alternative explanation (Wolff and Martarelli, 2020).

Interestingly, the pattern of change in pupil dilation we observed can also be tentatively interpreted in this vein. Research on exploration and exploitation behavior has identified pupil size as an indirect proxy for locus coeruleus (LC) activity (e.g., Gilzenrat et al., 2010). While exploration behavior seems to be associated with an increase in pupil diameter, exploitation is associated with decreased pupil size (Jepma and Nieuwenhuis, 2011). Thus, the constriction of the pupil during the first two blocks might capture exploitation behavior, while the re-enlargement might be a sign of exploration behavior, potentially triggered by rising boredom and leading to less task engagement and more errors. However, these interpretations should be made cautiously due to the non-differentiating measurement of pupil size between stimulus-evoked and baseline measures and because pupil dilation does not reflect one single psychological process. In the future, it may be helpful to analyze stimulus-evoked pupil diameter changes specific to Go and No-Go trials to distinguish phasic from tonic changes in pupil diameter.

Our results demonstrate the need to carefully examine different indicators of self-control. Interestingly, even though all indicators seemed to show similar declining trajectories, statistical analysis revealed that those measures were uncorrelated. One explanation could be the apparent high intra- and interindividual variability (Figures 1, 3). One approach to decrease potentially undesired variability would be the use of a gamified task to uphold motivation (Friehs et al., 2020). However, this variability can also be a source of information. While most subjects showed a decrease in inhibitory performance, subjective vitality, and pupil size, this decrease was not linear for all participants and some even showed increased or constant trajectories. This means that individuals who showed a decrease in performance did not necessarily experience a decrease in subjective vitality or pupil dilation. This again does not fit with the assumption of a depletable resource, namely that applying inhibitory control would generally weaken this capacity and lead to feelings of “depletion.” Rather, it fits with current empirical findings that this effect is highly individual and specific to the participant (Hagger et al., 2016).

## Conclusion

The present study was the first to investigate the temporal dynamics and associations of self-control processes by simultaneously assessing subjective, behavioral and psychophysiological measures in varying self-control demanding tasks. By showing a disconnect between these measures, we demonstrated that the interpretation of self-control exertion depends at least partially on the conceptualization of self-control. In addition to the theoretical advancements, this study thus provides methodological and meta research contributions. Future self-control research should place more emphasis on the theoretical conceptualization of self-control as a basis for a consistent derivation of the operationalized measures and tasks. The results obtained must first be interpreted consistently at the level of the operationalized variable. Only then a careful, but not overgeneralizing, retranslation of the measured findings to the authors’ conceptualization of self-control should take place.

## Data Availability Statement

The datasets presented in this study can be found in online repositories. The names of the repository/repositories and accession number(s) can be found below: https://osf.io/z4svm/.

## Ethics Statement

Ethical review and approval was not required for the study on human participants in accordance with the local legislation and institutional requirements. The patients/participants provided their written informed consent to participate in this study.

## Author Contributions

RB and ST developed the experimental design and carried out the data collection. ST performed data analysis. CE, WW, ST, and RB wrote the main manuscript text. All authors read and approved the final manuscript.

## Conflict of Interest

The authors declare that the research was conducted in the absence of any commercial or financial relationships that could be construed as a potential conflict of interest.

## Publisher’s Note

All claims expressed in this article are solely those of the authors and do not necessarily represent those of their affiliated organizations, or those of the publisher, the editors and the reviewers. Any product that may be evaluated in this article, or claim that may be made by its manufacturer, is not guaranteed or endorsed by the publisher.

## Footnotes

1 Crucially, one can also think of an experimental condition that is more boring than the respective control condition, further attesting to the need to control for boredom in future research.
